# Cellular inhibitor of apoptosis protein 2 promotes the epithelial-mesenchymal transition in triple-negative breast cancer cells through activation of the AKT signaling pathway

**DOI:** 10.18632/oncotarget.20227

**Published:** 2017-08-12

**Authors:** Su Ji Jo, Pil-Gu Park, Hye-Ran Cha, Sung Gwe Ahn, Min Jung Kim, Hyemi Kim, Ja Seung Koo, Joon Jeong, Jeon Han Park, Seung Myung Dong, Jae Myun Lee

**Affiliations:** ^1^ Department of Microbiology and Immunology, Yonsei University College of Medicine, Seoul, Republic of Korea; ^2^ BK21 PLUS Project for Medical Science, Yonsei University College of Medicine, Seoul, Republic of Korea; ^3^ Breast Cancer Center, Department of Surgery, Gangnam Severance Hospital, Yonsei University College of Medicine, Seoul, Republic of Korea; ^4^ Department of Pathology, Yonsei University College of Medicine, Seoul, Republic of Korea; ^5^ Research Institute, National Cancer Center, Goyang, Republic of Korea; ^6^ IMK Bio-Convergence R&D Center, International Vaccine Institute, Seoul, Republic of Korea

**Keywords:** triple-negative breast cancer, cellular inhibitor of apoptosis protein 2, epithelial-mesenchymal transition, AKT signaling pathway

## Abstract

Triple-negative breast cancer (TNBC) represents approximately 10–17% of all breast cancers, and patients with TNBC show a poorer short-term prognosis than patients with other types of breast cancer. TNBCs also have a higher tendency for early distant metastasis and cancer recurrence due to induction of the epithelial-mesenchymal transition (EMT). Several recent reports have suggested that inhibitor of apoptosis (IAP) proteins function as regulators of the EMT. However, the roles of these proteins in TNBC are not clear. Accordingly, we investigated the roles of cIAP2 in TNBC. Among eight *IAP* genes, only *cIAP2* was upregulated in TNBC cells compared with that in other breast cancer subtypes. Analysis of TMAs revealed that expression of cIAP2 was upregulated in TNBCs. *In vitro* studies showed that cIAP2 was highly expressed in TNBC cells compared with that in other types of breast cancer cells. Furthermore, silencing of cIAP2 in TNBC cells induced mesenchymal-epithelial transition (MET)-like processes and subsequently suppressed the migratory ability and invasion capacity of the cells by regulation of Snail through the AKT signaling pathway. In contrast, ectopic expression of cIAP2 in luminal-type breast cancer cells induced activation of the AKT signaling pathway. These results collectively indicated that cIAP2 regulated the EMT in TNBC via activation of the AKT signaling pathway, contributing to metastasis in TNBC. Our study proposes a novel mechanism through which cIAP2 regulates the EMT involving AKT signaling in TNBC cells. We suggest that cIAP2 may be an attractive candidate molecule for the development of targeted therapeutics in the future.

## INTRODUCTION

Breast cancer is currently one of the most common cancers. Worldwide, approximately 22% of new cancers are diagnosed as breast cancer, and the number of patients with breast cancer has reached about 3.9 million. In women, breast cancer is a leading cause of mortality, with approximately 520,000 annual deaths [1]. With the recent success of therapy based on molecular targeted drugs against breast cancer [2], further improvements in the efficacy of anticancer treatments are expected. However, there are no cure-all cancer therapies. Many types of breast cancers do not respond to current treatments, making the effective treatment of cancer difficult.

Clinically, breast cancer is usually categorized based on the expression of specific receptors, including estrogen receptor (ER), progesterone receptor (PR), and human epidermal growth factor receptor 2 (HER2)/neu (also known as ERBB2). Cancers expressing none of these three receptors are referred to as triple-negative breast cancers (TNBCs), which account for 10–17% of all breast cancers [3]. TNBCs show a relatively poorer prognosis compared with other types of breast cancer and often cannot be subjected to current targeted therapies due to the absence of receptors, which can act as therapeutic target molecules. Additionally, TNBCs are known to be associated with a higher tendency of progression to metastatic diseases, the major cause of breast cancer-related death [4]. The lack of efficient targeted therapy and higher potential for metastatic progression of TNBCs need to be overcome in order to improve breast cancer therapies. To conquer breast cancer, novel strategies based on molecular mechanisms associated with TNBC will be necessary.

Cancer metastasis is a multicellular process involving a series of steps from primary cancer to metastatic malignancy at distal sites. During metastasis, cancer cells in primary tumors must change from the epithelial phenotype to the mesenchymal phenotype in order to penetrate into the basement membrane, a process called the epithelial-mesenchymal transition (EMT) [5]. Many studies have shown that transcription factors, including Snail/Slug, Twist, and Zeb family proteins, govern the overall EMT process by regulation of various structural and adhesion/junction molecules [6]. Because the EMT is a key contributor to the metastatic process, its regulatory mechanisms should be well organized and controlled. Although various transcription factors and their target genes have been extensively studied in the EMT processes, signaling molecules associated with the EMT are relatively poorly understood. Recently, several studies have reported novel molecules with EMT regulatory functions [7-9].

Inhibitor of apoptosis proteins (IAPs), also known as baculovirus IAP repeat (BIR) domain-containing proteins (BIRCs), are a protein family including eight proteins commonly possessing BIR domains, including BIRC1 (NAIP), BIRC2 (cIAP1), BIRC3 (cIAP2), BIRC4 (XIAP), BIRC5 (Survivin), BIRC6 (BRUCE), BIRC7 (Livin), and BIRC8 (ILP2) [10]. IAPs many function as E3 ligases owing to their C-terminal RING domains and are able to degrade proteins by linking them to ubiquitin molecules [11]. Because XIAP, cIAP1, and cIAP2 are directly or indirectly related to the apoptosis pathway through inhibition of caspases, many drugs for cancer therapy have focused on inhibition of IAPs [12].

Recently, several reports have suggested the possibility of another function of IAPs as regulators of the EMT [13]. However, the function of IAPs related to the EMT process and cancer metastasis is still unclear. XIAP and cIAP2 are thought to be positive regulators of the EMT [14-16]. In contrast, one study showed that XIAP and cIAP1 inhibit the EMT by inducing Rac1 degradation [17]. Thus, because of the inconsistencies in clinical analysis of IAPs in cancer prognosis, further analyses are needed to determine the roles of IAPs in cancer progression and metastasis.

In this study, we evaluated the expression patterns of IAPs in several breast cancer cell lines. Subsequently, we examined the role of cIAP2 in TNBC and the EMT. Our findings provided important insights into the mechanisms through which cIAP2 modulates the EMT in TNBC.

## RESULTS

### cIAP2 was highly expressed in TNBC

**Figure 1:**
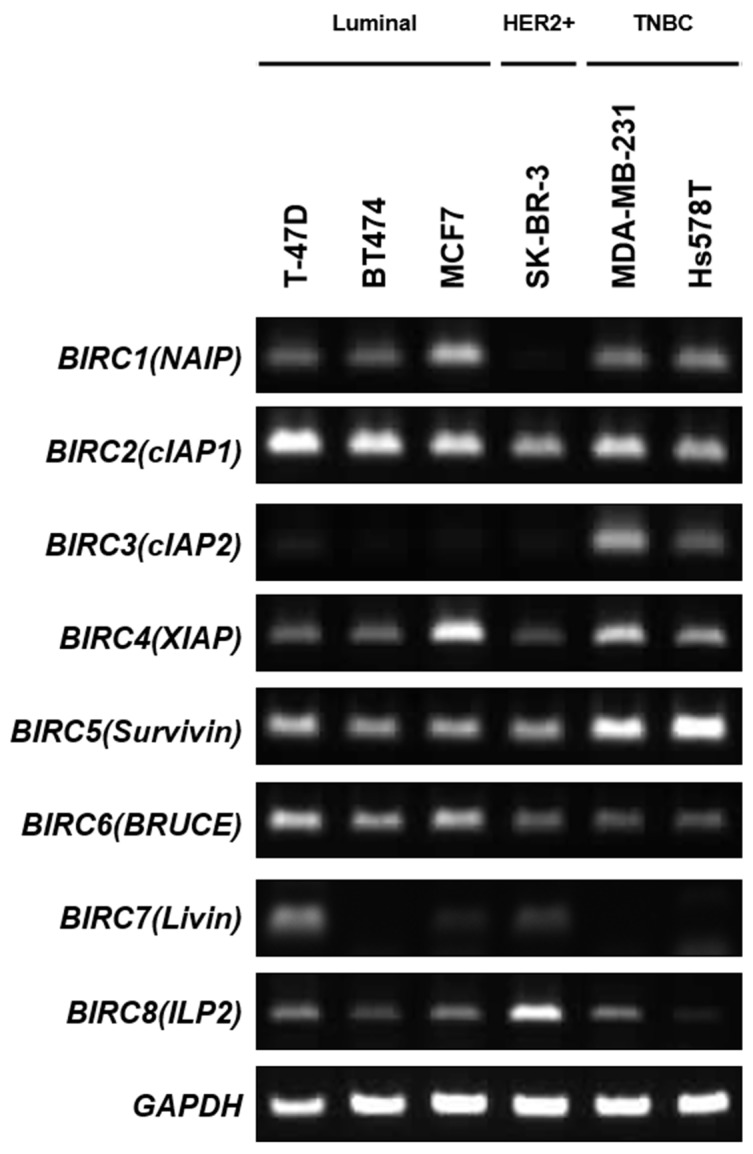
Analysis of IAP expression in breast cancer cells mRNA expression of IAPs was examined in luminal-type breast cancer cells (T-47D, BT474, MCF7), HER2-positive breast cancer cells (SK-BR-3), and triple-negative breast cancer cells (MDA-MB-231, Hs 578T).

To investigate the relevance of IAP expression in TNBC, we assessed mRNA expression levels for all IAP members in luminal-type (T-47D, BT474, MCF7), HER2-positive-type (SK-BR-3), and triple-negative (MDA-MB-231 and Hs 578T) breast cancer cell lines (Supplementary Table 1) [18-20]. Among the eight IAP genes evaluated in this study (*BIRC1–8*), only cIAP2 (*BIRC3*) was highly expressed in TNBC cells compared with that in other breast cancer subtypes, while other IAP members were detected in various breast cancer cells, irrespective of their subtypes (Figure 1).

**Figure 2:**
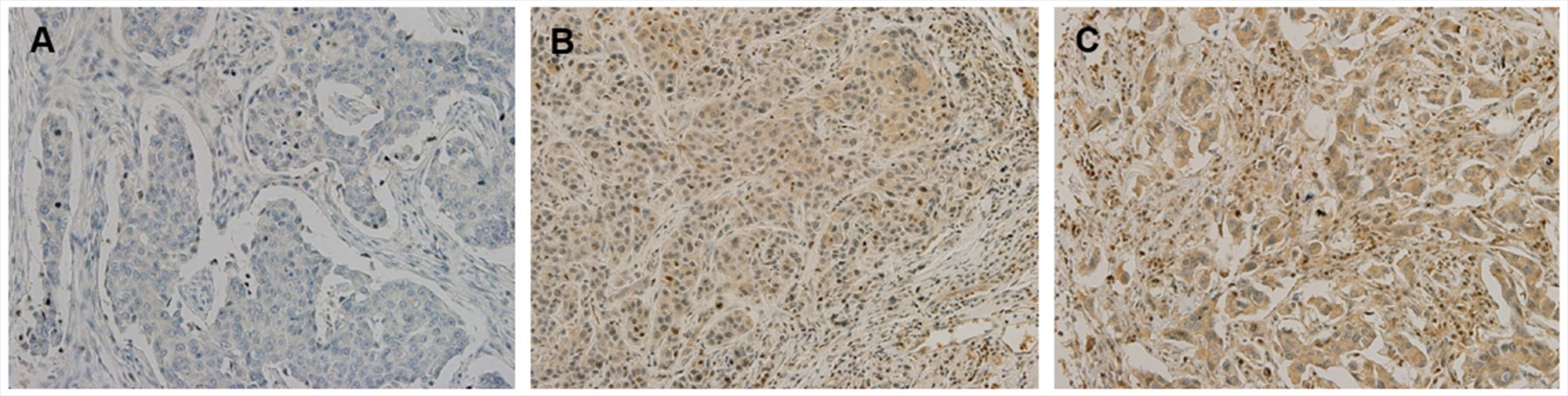
Immunohistochemical analysis of cIAP2 in triple-negative breast cancer tissues Expression of cIAP2 in triple-negative breast cancer tissue was evaluated in high-power fields (200×). **(A)** Negative for cIAP2. **(B)** 1+ for cIAP2. **(C)** 2+ for cIAP2. cIAP2 was generally observed in the cytoplasm in triple-negative breast cancer tissue.

To determine the expression level of cIAP2 in breast cancers tissues, we performed TMA analysis using IHC staining. When classified according to the degree of cIAP2 expression (Figure 2 and Supplementary Table 2), about 96% of patients with TNBC showed positive expression of cIAP2 in cancer tissues, whereas only 4% of TNBC tissues showed no cIAP2 expression (Table 1), suggesting that cIAP2 may play a role in TNBC.

**Table 1:** Comparison of cIAP2 expression in triple-negative breast cancer tissue microarrays using immunohistochemistry (IHC)

Interpretation	TNBC TMA (N=100)		
	Quick score	Frequency	Percent
Negative (0)	0	4	4%
	1	0	
	2	0	
Weak/Moderate (1+)	3	20	52%
	4	13	
	5	19	
Strong (2+)	6	38	44%
	7	6	
	8	0	

The cIAP2-positive status was assigned for scores “1+” and “2+.”

### cIAP2 expression was related to EMT-associated gene expression

**Figure 3:**
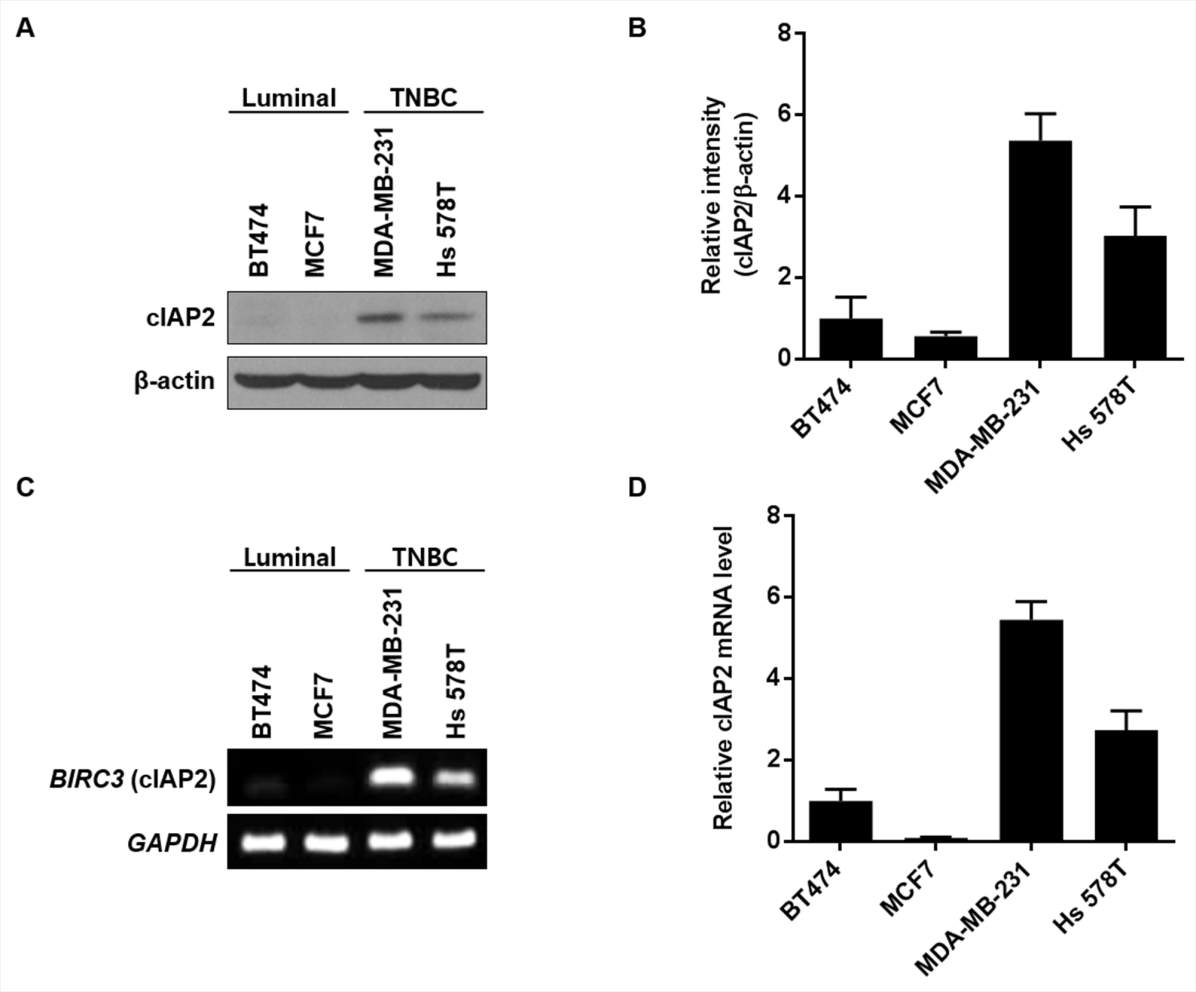
cIAP2 expression in breast cancer cell lines Expression of cIAP2 was examined on luminal-type (BT474, MCF7) and triple-negative (MDA-MB-231, Hs 578T) breast cancer cells. **(A)** Western blotting was performed to determine the protein expression level of cIAP2. β-Actin was used as a loading control. **(B)** Graphical representation of western blot band intensities quantified using Image J (n = 3). Expression of *cIAP2* mRNA was examined by **(C)** RT-PCR and **(D)** quantitative real-time PCR. For quantification of the results in (B) and (D), the cIAP2 protein and mRNA level for BT474 cells was set to 1. Values represent the means ± standard deviations.

To confirm the molecular meaning of the TMA results, we next assessed mRNA and protein levels of cIAP2 in luminal-type breast cancer cell lines (BT474 and MCF7; non-TNBC) and TNBC cell lines (MDA-MB-231 and Hs 578T). In accordance with IHC analysis of breast cancer TMA in Table 1, cIAP2 was highly expressed in TNBC cell lines but barely detected in luminal-type cell lines at both the protein (Figure 3A and 3B) and mRNA (Figure 3C and 3D) levels.

**Figure 4:**
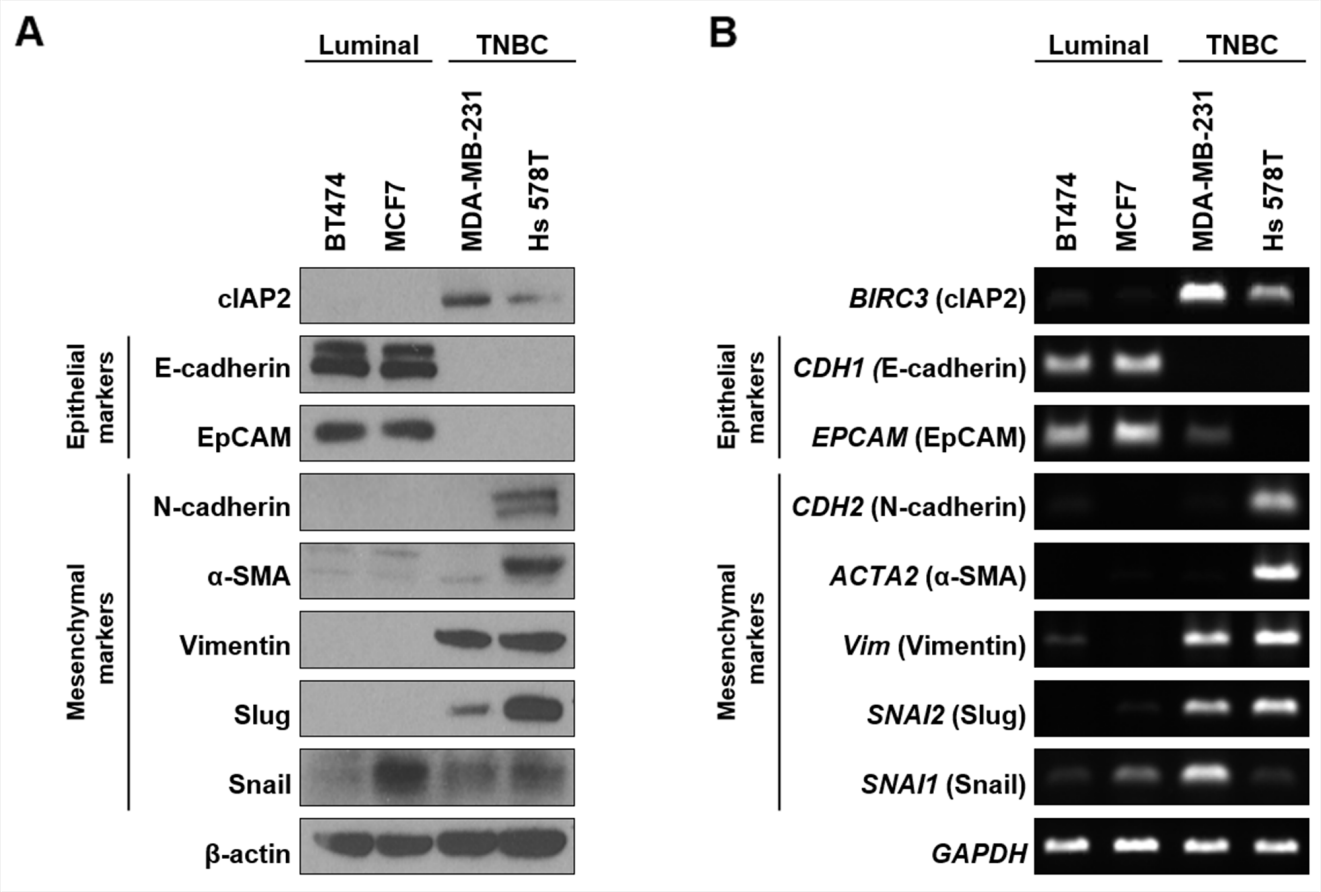
Comparison of cIAP2 expression and EMT-associated molecules in breast cancer cells Expression of cIAP2 and EMT-associated molecules were examined in luminal-type (BT474, MCF7) and triple-negative (MDA-MB-231, Hs 578T) breast cancer cells at **(A)** protein and **(B)** mRNA levels. β-Actin and GAPDH were used as loading controls.

Our data for TMA, qPCR, and IHC analyses showed strong correlations between cIAP2 expression and TNBC. Thus, we hypothesized that cIAP2 may play important roles in regulating the clinical features of TNBCs, including poor prognosis and high metastatic tendency. Therefore, we investigated the possibility that cIAP2 may regulate the metastatic characteristics of breast cancers by detection of epithelial/mesenchymal markers known to be closely related to metastatic progression. At both the protein and mRNA levels, epithelial markers were detected in luminal-type cell lines; in contrast, mesenchymal markers were primarily expressed in TNBC cell lines (Figure 4A and 4B). Next, wound healing assays were performed to determine the effects of cIAP2 expression on the metastatic phenotype. The results showed far higher migration capacity in TNBC cell lines compare with that in luminal-type cell lines (Supplementary Figure 1).

**Figure 5:**
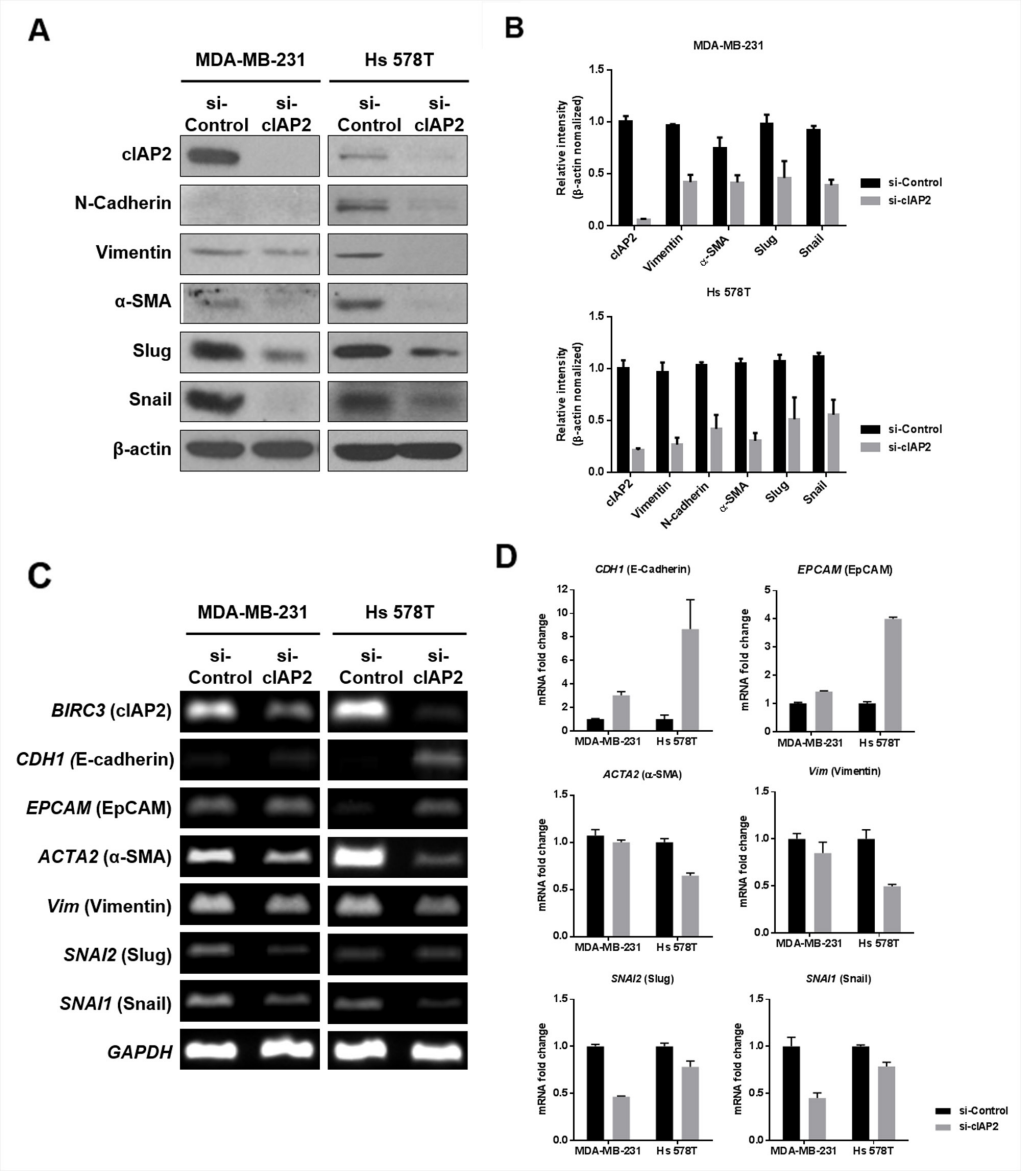
Analysis of EMT-associated molecules in TNBC cells (MDA-MB-231 and Hs 578T) with cIAP2 silencing **(A)** Expression of EMT-associated mesenchymal markers, including N-cadherin, vimentin, α-SMA, Snail, and Slug, was determined at the protein level in MDA-MB-231 and Hs 578T cells transfected with si-cIAP2. β-Actin was used as a loading control. **(B)** Graphical representation of western blot band intensities quantified using Image J (n = 3). **(C** and **D)** Expression of EMT-associated markers, including *CDH1*, *EPCAM*, *ACTA2*, *Vim*, *SNAI1*, and *SNAI2*, was determined at the mRNA level in MDA-MB-231 and Hs 578T cells transfected with si-cIAP2 by (C) RT-PCR and (D) quantitative real-time PCR. Values represent the means ± standard deviations.

Based on epithelial/mesenchymal marker expression patterns and migratory capacity (Figure 4 and Supplementary Figure 1), we speculated that cIAP2 may function as a regulator of the EMT. To verify this, we evaluated changes in epithelial and mesenchymal markers after silencing of *cIAP2* mRNA in TNBC cell lines. Notably, all of the mesenchymal markers we tested were reduced by cIAP2 knockdown at both the protein and mRNA levels (Figure 5A-5C). In contrast, mRNA of epithelial markers, including *CDH1* (encoding E-cadherin) and *EPCAM* (encoding EpCAM) were obviously increased in cIAP2-knockdown cells (Figure 5C and 5D). These changes in mesenchymal to epithelial markers by cIAP2 knockdown indicated that cIAP2 was a positive regulator of the EMT process.

### cIAP2 knockdown suppressed cell migration and invasion in breast cancer cell lines

**Figure 6:**
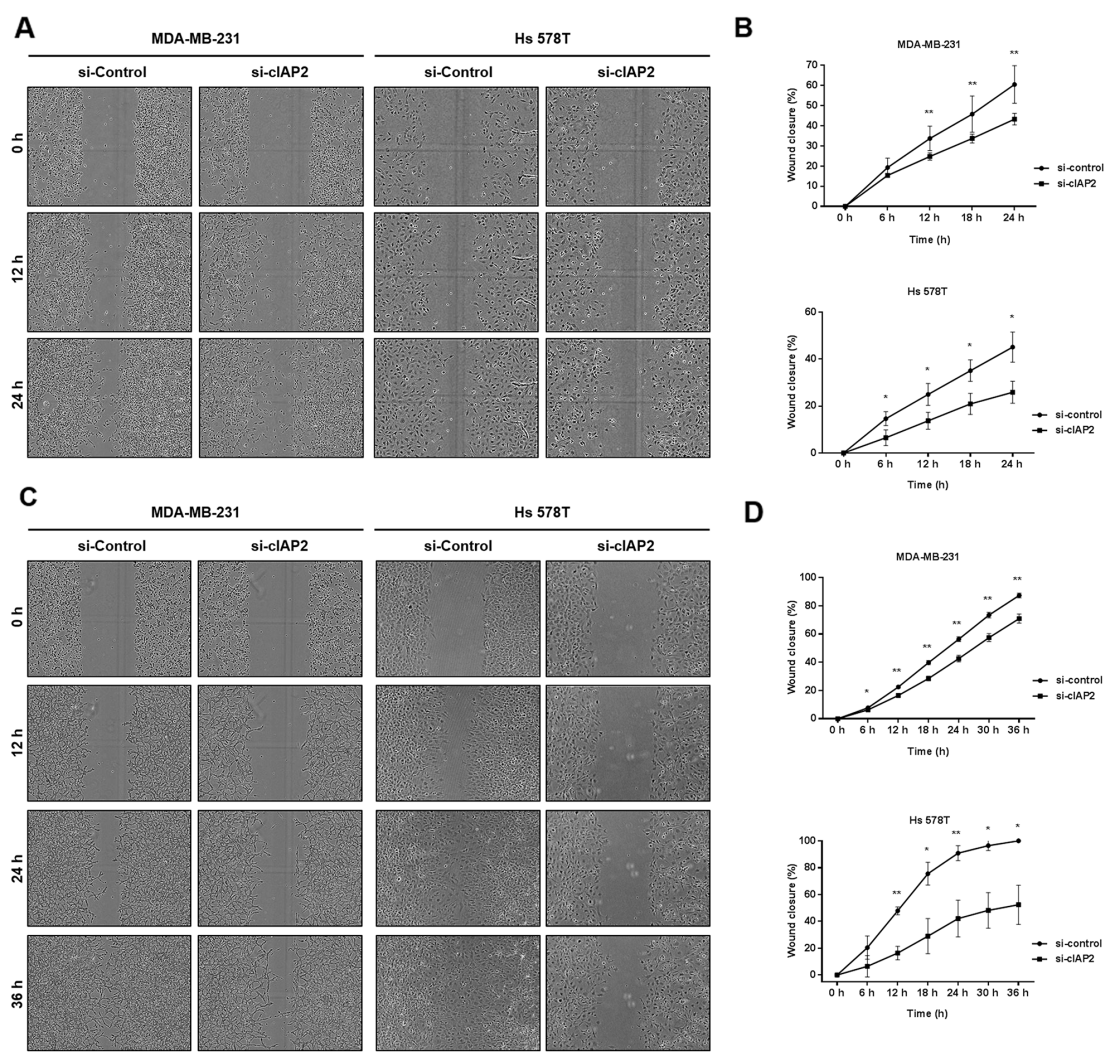
Silencing of cIAP2 suppressed the migration and invasion ability of TNBC cells (MDA-MB-231 and Hs 578T) **(A)** Migration of MDA-MB-231 (si-control and si-cIAP2) and Hs 578T (si-control and si-cIAP2) cells was examined by wound healing assay. **(C)** Invasion capacity of MDA-MB-231 (si-control and si-cIAP2) and Hs 578T (si-control and si-cIAP2) cells was examined by scratch wound invasion assay. The wound gap was photographed every 6 h. The initial wound width was 700 μm (10× magnification). **(B** and **D)** Wound closure (%) was measured using IncuCyte ZOOM software (n = 5). Values represent the means ± standard deviations. *P < 0.05, **P < 0.01, ***P < 0.001.

During the EMT process, changes in epithelial/mesenchymal features of cells lead to migration and invasion, which are essential for cancer metastasis [21]. Thus, we assessed the migration and invasion capacities of cIAP2-knockdown TNBC cells. In wound healing assays, control MDA-MB-231 and Hs 578T cells showed high migratory capacity, consistent with the results shown in Supplementary Figure 1; however, significantly attenuated cell migration was observed in both cell lines with cIAP2 knockdown (Figure 6A and 6B). The wounds were then filled with Matrigel to mimic the extracellular matrix in order to assess invasiveness. Our analysis showed that invasiveness was greatly inhibited in cIAP2-knockdown cells (Figure 6C and 6D). Thus, cIAP2 played critical roles in determining the cell migration and invasion capacity in TNBC cells.

One of the molecular functions of cIAP2 is inhibition of cell death [11], which may affect cell migration and invasion. However, among IAPs, the major inhibitory molecule of apoptosis is thought to be XIAP, and cIAP2 may have a relatively minor role in the regulation of apoptosis, despite its binding affinity to caspase [22, 23]. To exclude the effects of cIAP2 on cell death in our measurement of cell migration and invasion, we also evaluated cell proliferation after cIAP2 knockdown; no significant difference was observed between si-scramble- and si-cIAP2-transfected cells (Supplementary Figure 2). Thus, the reduced migratory/invasive capacity of cIAP2-knockdown cells was assumed to be mostly due to inhibition of the EMT.

### The AKT signaling pathway was related to cIAP2-mediated induction of the EMT

**Figure 7:**
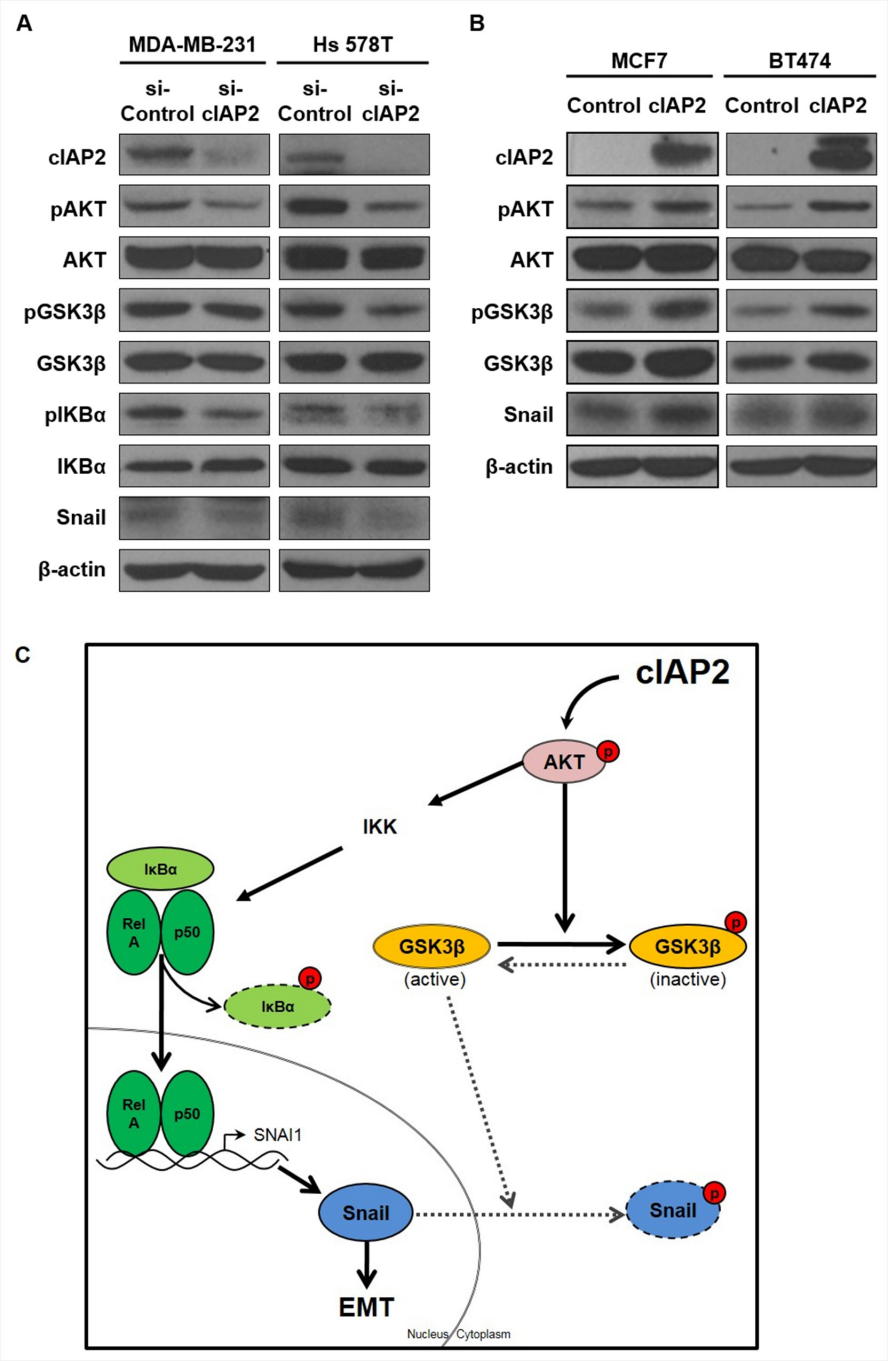
cIAP2 promoted the EMT through the AKT signaling pathway The expression of AKT signaling pathway-associated molecules was analyzed in **(A)** cIAP2-silenced TNBC cells and **(B)** luminal-type breast cancer cells ectopically expressing cIAP2. β-Actin was used as a loading control. **(C)** Proposed model for the molecular mechanisms through which cIAP2 induced the EMT. The bold lines represented an effect that was reinforced by cIAP2, whereas the dotted lines represented an effect that was weakened by cIAP2.

Next, we attempted to investigate the molecular mechanisms of cIAP2-mediated EMT induction. Several signaling pathways related to the EMT process were evaluated (data not shown), and we found that the AKT signaling pathway was significantly altered following cIAP2 manipulation (Figure 7). In previous reports, the AKT signaling pathway was shown to induce the EMT process through inhibitory phosphorylation of GSK3β, a negative regulator of the EMT-related transcription factor Snail [24, 25]. Thus, we then evaluated the expression of AKT-associated signaling molecules in cIAP2-knockdown TNBC cells. Western blot analysis showed that phosphorylation of AKT was attenuated by silencing of cIAP2, leading to reduced phosphorylation of GSK3β and subsequent destabilization of Snail (Figure 7A). As a consequence of reduced GSK3β phosphorylation, we observed reduced expression of Snail at the protein level (Figure 5A and 5B). Additionally, we analyzed the expression of AKT-associated signaling molecules in cIAP2-transfected MCF7 and BT474 cells. Western blot analysis showed that phosphorylation of AKT was increased by ectopic expression of cIAP2, leading to increased GSK3β phosphorylation and subsequent stabilization of Snail protein (Figure 7B).

Interestingly, *SNAI1* expression was also decreased by silencing of cIAP2 (Figure 5C and 5D). Nuclear factor kappa B (NF-κB) is a well-known transactivator of *SNAI1* [26] and a target of AKT regulation [27]. Therefore, we next evaluated the phosphorylation of IκBα, which acts as an inhibitor of NF-κB activation. In western blot analysis, we found reduced phosphorylation of IκBα by silencing of cIAP2 (Figure 7A).

Thus, our data collectively suggested that cIAP2 induced the EMT through activation of both AKT/GSK3β and AKT/NF-κB signaling pathways (Figure 7C).

## DISCUSSION

IAPs are proto-oncogenes involved in the regulation of tumor progression, metastasis, cell survival, and migration. In particular, cIAP2 is upregulated in colorectal cancer, mucosa associated lymphoid tissue lymphoma, acute myeloid leukemia, and lung cancer and is associated with poor tumor prognosis [10]. However, no reports have described the clinical significance of cIAP2 in breast cancers.

Although cIAP2 has been reported to be upregulated in several types of cancer, the roles of IAPs in the EMT are still unclear [14, 15, 17, 28, 29]. IAPs share high homology and show functional redundancy; however, each IAP may induce different effects in EMT regulation. In the current study, our findings supported that cIAP2 functioned as a positive regulator of the EMT in TNBC. Despite these findings, it is still unclear whether other IAPs also promote the EMT process, although their expression levels did not differ among cell lines showing low and high migratory capacity.

In this study, we verified that cIAP2, which was upregulated in TNBC compared with other breast cancer subtypes, induced the EMT in TNBC cells via activation of the AKT signaling pathway. Several reports of IAP-mediated EMT induction have shown that this mechanism involves the regulation of small Rho GTPases [14, 16, 17, 30, 31]. Gradients of Rho GTPase proteins are associated with cell polarity and mobility, which are critical factors involved in the EMT process [32]. Because the EMT process involves various cellular changes, adjustment of major transcription factors, such as Snail, Twist, and Zeb family proteins, is also necessary. Accordingly, AKT signaling and Snail regulation by cIAP2 could be the main mechanism mediating the EMT process.

Dysregulation of AKT signaling in breast cancer has been extensively reported in previous studies. The activating mutation of PIK3CA is frequently observed in breast cancers, particularly luminal-type cancer, which leads to hyperactivation of the AKT signaling pathway [33-35]. The luminal-type breast cancer cell lines used in our study (MCF7 and BT474 cells) had mutations in PIK3CA [19, 36], whereas TNBC cell lines (MDA-MB-231 and Hs 578T cells) had wild-type PIK3CA. In our experiment, BT474 cells showed highly activated AKT signaling despite weak expression of cIAP2 (data not shown); this could be explained by the presence of mutated PIK3CA. In the case of MCF7 cells, relatively low levels of phospho-AKT were detected (data not shown), consistent with previous studies showing that some luminal-type breast cancer cell lines, such as MCF7 cells, show weak activation of AKT signaling despite PIK3CA mutations; the authors speculated that this effect may be related to the function of another AKT regulator. Notably, in our study, cIAP2 was expressed only in TNBC, and luminal-type breast cancer was negative for cIAP2. Therefore, cIAP2 may be responsible for the induction of AKT signaling in TNBCs, whereas AKT activation in luminal-type breast cancer was mainly affected by the status of PIK3CA and other factors, independent of cIAP2 expression.

The AKT signaling pathway has been shown to positively influence EMT progression [37]. The mechanisms of AKT-mediated EMT induction can be summarized as follows. First, activated AKT phosphorylates GSK3β (inhibitory phosphorylation) and subsequently prohibits the phosphorylation of Snail, leading to its degradation [25]. Second, activated AKT can also phosphorylate and activate IKK, which triggers nuclear localization of NF-κB. Then, NF-κB activates the transcription of *SNAI1* [38]. Consistent with these findings, we observed reduced Snail expression at both the protein and mRNA level, following cIAP2 knockdown, suggesting that cIAP2 induced the EMT by both transcriptional and post-translational regulation of Snail through induction of AKT signaling.

Slug is another transcription factor with important roles in the EMT process [39]. In this study, we observed changes in *SNAI2* (Slug) as well as *SNAI1* (Snail) at both the mRNA and protein levels after cIAP2 manipulation, suggesting the possibility of Slug regulation by AKT signaling, similar to the mechanism described for Snail regulation. Indeed, post-translational regulation of Slug by AKT/GSK3β signaling has recently been described [39]. Additionally, some reports have shown that Slug is regulated by the NF-κB signaling pathway [40, 41]. However, the roles of AKT in regulation of *SNAI2* have not yet been clarified. Further studies are needed to better establish the roles of the AKT signaling pathway in the EMT process.

The NF-κB signaling pathway is involved in many biological processes, including cell proliferation, cell differentiation, immune responses, and apoptosis, and the dysregulation of NF-κB is involved in many diseases, including inflammatory/autoimmune disease, tissue malformation, and tumorigenesis [42]. cIAP1 and cIAP2 have been reported to be essential for NF-κB signal transduction via K63-linked ubiquitination of RIP1 [11, 12]. At the same time, cIAP1 and cIAP2 are also targets of NF-κB-transactivation, suggesting the involvement of positive feedback in the regulation of cIAP1/2 expression [43]. As shown in our study, cIAP2 activated AKT signaling, supporting the involvement of another feedback loop in the regulation of cIAP1/2 expression as AKT signaling can also activate NF-κB. Although more in-depth studies are required, our results provided insights into a novel signaling pathway and could improve our understanding of NF-κB signal transduction.

We observed a clear, significant effect of cIAP2 knockdown on the EMT process in cIAP2-positive TNBC cell lines. However, ectopic expression of cIAP2 in breast cancer cell lines, which rarely expressed cIAP2, did not show meaningful changes in the EMT process, except activation of the AKT signaling pathway. This may due to the masking effect of ER and/or PR in breast cancer cells. Consistent with our study, most cIAP2-negative breast cancer cell lines are luminal type (expressing ER and/or PR), and some reports have suggested that ER and/or PR repress EMT progression [44-51], although these findings are controversial [52-55]. In accordance with this, it is possible that EMT progression is eventually prohibited by ER and/or PR signaling in cIAP2-negative breast cancer cells, despite ectopic expression of cIAP2. To determine the exact mechanisms mediating this process, more in-depth studies are required.

Recently, several small molecule inhibitors of IAPs (including diablo IAP-binding mitochondrial protein-mimicking molecules [SMAC mimetics]) have been developed, some of which have entered phase II trials as potential cancer therapies [56]. The major evidence supporting the use of SMAC mimetics is induction of apoptosis by inhibition of IAPs, which can drive cancer cell death. Thus, based on our findings, it is possible that SMAC mimetics also attenuate EMT progression via inhibition of cIAP2, eventually acting to block cancer metastasis. Therefore, SMAC mimetics, as therapeutics for both cell death induction and EMT inhibition, may be novel candidates for the treatment of TNBC.

Our current findings revealed that cIAP2 was highly expressed in TNBC compared with other types of breast cancer, both *in vivo* and *in vitro*. Additionally, we found that cIAP2 regulated the EMT via the AKT signaling pathway. Our data suggested that cIAP2 could be an attractive candidate for development of targeted therapeutics and a potential diagnostic marker for predicting tumor metastasis.

## MATERIALS AND METHODS

### Cell culture

Human breast cancer cell lines, including T-47D, BT474, MCF7, SK-BR-3, MDA-MB-231, and Hs 578T cells, were purchased from American Type Culture Collection (ATCC, Manassas, VA, USA) and grown in accordance with ATCC recommendations. Cell culture medium was supplemented with 10% heat-inactivated fetal bovine serum (FBS; Hyclone, Logan, UT, USA) and 10 U/mL penicillin-streptomycin (Hyclone). The cells were cultivated at 37°C in a humidified atmosphere containing 5% CO_2_.Characteristics of the breast cancer cell lines used in this study are described in Supplementary Table 1. All cell lines used in this study were authenticated by short tandem repeat profiling at the Research Institute of National Cancer Center (Goyang, Gyeonggi-do, Republic of Korea).

### Construction of cIAP2-siRNA and cIAP2 expression vector

For knockdown of cIAP2 (NM_001165) mRNA, the following sequences were used: si-cIAP2, 5′-GGAGAGAAUUAUAGACUAGUCAATG-3′; si-Control (nonspecific oligo), 5′-CGUUAAUCGCGUAUAAUACGCGUA-3′ (IDT, Coralville, IA, USA). MDA-MB-231 and Hs 578T cells were transfected with cIAP2-siRNA or control-siRNA at 50% cell confluence using Lipofectamine RNAi MAX (Invitrogen, Green Island, NY, USA) according to the manufacturer’s instructions. The optimal concentrations of siRNA for gene silencing were 50 nM for MDA-MB-231 cells and 25 nM for Hs 578T cells.

For ectopic expression of cIAP2, the pRK5-Flag-cIAP2 plasmid construct was purchased from Addgene (Cambridge, MA, USA). The pRK5-Flag vector was used as a control. The plasmids were transfected into breast cancer cells using Lipofectamine 2000 (Invitrogen), according to the manufacturer’s instructions.

### Cell proliferation assay

Cell proliferation rates were measured using a Cell Counting Kit-8 (CCK-8; Dojindo Molecular Technologies, Inc., Rockville, MD, USA) according to the manufacturer’s instructions. Briefly, cIAP2-silenced TNBC cells were plated in 96-well microplates at a density of 3 × 10^3^ cells/well for MDA-MB-231 cells and 1 × 10^3^ cells/well for Hs 578T cells. Next, 10 μL CCK-8 solution (2-[2-methoxy-4-nitrophenyl]-3-[4-nitrophenyl]-5-[2, 4-disulfophenyl]-2H-tetrazolium) was added to each well. The plates were incubated at 37°C in an incubator for 3 h, and the absorbance at 450 nm was measured using an Epoch Microplate Spectrophotometer (BioTek Instruments, Inc., Winooski, VT, USA).

### Western blot analysis

Cell lysates were prepared in RIPA buffer (50 mM Tris-Cl [pH 7.5], 150 mM NaCl, 1% NP-40, 0.5% sodium deoxycholate, 0.1% sodium dodecyl sulfate [SDS]) supplemented with protease inhibitors. The protein concentration was determined using a BCA assay kit (Thermo Fisher, Waltham, MA, USA). Whole-cell extracts were boiled in 4× SDS sample buffer (2% SDS, 50 mM Tris-HCl [pH 6.8], 0.4 mM ethylenediaminetetraacetic acid [EDTA, pH 8.5], 10% glycerol, 0.002% pyronin Y) supplemented with 2-mercaptoethanol. The denatured protein was separated by SDS polyacrylamide gel electrophoresis (SDS-PAGE) on 10% gels and transferred to 0.45-μm nitrocellulose membranes (Bio-Rad, Hercules, CA, USA) by semidry transfer. The membranes were washed with Tris-buffered saline containing 0.05% Tween 20 (TBS-T) and blocked for 1 h in TBS-T containing 5% skim milk. The membranes were then incubated with primary antibodies diluted with TBS-T at 4°C overnight and then washed three times with TBS-T. The membranes were incubated with horseradish peroxidase (HRP)-conjugated secondary antibodies diluted in TBS-T containing 5% skim milk at room temperature for 90 min, followed by five washes with TBS-T. Finally, protein bands were visualized using WesternBright ECL Chemiluminescent HRP Substrate (Advansta, Menlo Park, CA, USA).

Primary antibodies were as follows: anti-cIAP2 (#3130; Cell Signaling Technology, Danvers, MA, USA), anti-E-cadherin (610181; BD Biosciences, Sparks, MD, USA), anti-epithelial cell adhesion molecule (EpCAM; ab71916; Abcam, Milton, Cambridge, UK), anti-N-cadherin (33-3900; Invitrogen), anti-α-smooth muscle actin (SMA; RB-9010-P; Invitrogen), anti-vimentin (M0725; Dako, Glostrup, Denmark), anti-Slug (#9585; Cell Signaling Technology), anti-Snail (#3895; Cell Signaling Technology), anti-phospho-AKT (Ser473; #4058; Cell Signaling Technology), anti-total AKT (#4691; Cell Signaling Technology), anti-phospho-glycogen synthase kinase (GSK) 3β (Ser9; #5558; Cell Signaling Technology), anti-GSK3β (#9315; Cell Signaling Technology), anti-phospho-inhibitor of kappa B alpha (IκBα; Ser32; #2859; Cell Signaling Technology), anti-IκBα (#9242; Cell Signaling Technology), anti-β-actin (AC-15; Sigma Aldrich, St. Louis, MO, USA).

### Immunohistochemical (IHC) staining

TNBC tissue microarray (TMA) slides (100 cases) were obtained from Gangnam Severance Hospital (Yonsei University, Seoul, Korea). The Institutional Review Board of Gangnam Severance Hospital approved this study in accordance with good clinical practice guidelines and the Declaration of Helsinki (3-2014-0239).

An OPTIVIEW Universal DAB kit (Ventana Medical Systems, Tucson, AZ, USA) was used for IHC staining. Slides were deparaffinized and rehydrated with sequential incubation in xylene, 100% to 70% ethanol, and distilled water. After antigen retrieval with cell conditioner 1 for 24 min at 100°C, slides were immersed with 3% H_2_O_2_ for 4 min at 37°C and probed with anti-cIAP-2 antibodies (NB100-56132; Novus International, St. Charles, MO, USA) for 16 min at 37°C. OptiView HQ Universal Linker and OptiView HRP Multimer were sequentially added to the slides for 8 min each. Subsequently, 3,3′-diaminobenzidine (DAB) substrate solution was added. Slides were counterstained with hematoxylin and bluing reagent. Digital images of slides with DAB staining were obtained using Leica Application Suite (LAS) Microscope Software (Leica Microsystems Inc., Buffalo Grove, IL, USA).

IHC staining for cIAP2 was classified as negative (0), 1+, or 2+ in high-magnification (200×) fields according to the intensity of cytoplasmic staining and using quantitative scoring methods [57]. The scoring methods used in this study are described in Supplementary Table 2. The cIAP2-positive status was assigned for scores 1+ and 2+. The interpretation of IHC staining was evaluated by a pathologist who had no information regarding the clinical outcomes.

### Scratch wound cell migration assay (wound healing assay)

To generate wounds on breast cancer cells, IBIDI culture inserts (IBIDI, Martinsried, Germany) were placed into wells of 24-well plates, and cells were added into the two reservoirs of the inserts. Cells were plated at 1.5 × 10^5^ BT474 cells/well, 1.2 × 10^5^ MCF7 cells/well, 4 × 10^4^ MDA-MB-231 cells/well, and 3 × 10^4^ Hs 578T cells/well. After incubation for 24 h in a 37°C CO_2_ incubator, the culture inserts were removed, and images of the wounds were taken at 0 and 24 h using an Olympus IX71 microscope (Olympus France; Rungis, France).

For kinetic analysis, cIAP2-silenced MDA-MB-231 cells (4 × 10^4^ cells/well) and Hs 578T (1 × 10^4^ cells/well) were seeded into a 96-well Essen ImageLock microplate (Essen BioScience, Ann Arbor, MI, USA) and incubated in a 37°C CO_2_ incubator for 24 h. The cell monolayer was scratched using a 96-well Wound Maker and then washed with phosphate-buffered saline (PBS) to remove the detached cells. Images of the wounds were automatically recorded at 6-h intervals for 24 h using IncuCyte ZOOM software.

### Scratch wound cell invasion assays

cIAP2-silenced MDA-MB-231 cells (4 × 10^4^ cells/well) and Hs 578T cells (1 × 10^4^ cells/well) were seeded into a 96-well Essen ImageLock microplate coated with diluted Matrigel (100 μg/mL) in cold culture medium, and cells were then incubated in a 37°C CO_2_ incubator for 24 h. The cells were scratched using a 96-well Wound Maker and then washed with PBS to remove detached cells. Next, 50 μL diluted Matrigel (8 mg/mL) in cold culture medium was added to each well, and cells were incubated in a 37°C CO_2_ incubator for 30 min. When the Matrigel solution was solidified, 100 μL cell culture medium was added to each well. Images of the wounds were automatically recorded at 6-h intervals for 48 h using IncuCyte ZOOM software.

### Reverse transcription polymerase chain reaction (RT-PCR) and quantitative real-time RT-PCR

RNA was extracted using TRIzol reagent (Invitrogen), and cDNA was synthesized using Oligo dT primers (Invitrogen) and SuperScript III Reverse Transcriptase (Invitrogen) according to the manufacturer’s instructions. Synthesized cDNAs were used as templates for subsequent PCR of *BIRC1*, *BIRC2*, *BIRC3*, *BIRC4*, *BIRC5*, *BIRC6*, *BIRC7*, *BIRC8*, *CDH1*, *CDH2*, *EPCAM*, *VIM*, *ACTA2*, *SNAI1*, *SNAI2*, and *GAPDH* genes*.* Primer sequences used in this study are described in Supplementary Table 3.

Quantitative real-time RT-PCR was carried out using a KAPA SYBR FAST qPCR kit (KaPa Biosystems, Wilmington, MA, USA), and amplification was performed on an ABI Prism 7000 detection system (Applied Biosystems, Waltham, MA, USA) according to the conditions recommended by the manufacturer. The experiments were performed in triplicate and normalized to the expression of hypoxanthine-guanine phosphoribosyltransferase (HPRT). The relative expression levels of the target genes were calculated by the 2^−ΔΔCt^ method.

### Statistical methods

Unpaired Student’s *t*-tests were used to determine significant differences between groups. All statistical analyses were performed using GraphPad Prism 6.0a software (GraphPad Software, La Jolla, CA, USA). Values represent means ± standard deviations (SDs). Differences with *P* values of less than 0.05 were considered significant.

## SUPPLEMENTARY MATERIALS FIGURES AND TABLES



## Abbreviations

EMT: epithelial-mesenchymal transition
MET: mesenchymal-epithelial transition
IAP: inhibitor of apoptosis protein
TNBC: triple-negative breast cancer
ER: estrogen receptor
PR: progesterone receptor
HER2: human epidermal growth factor receptor
TMA: tissue microarray
PBS: phosphate-buffered saline
SDS: sodium dodecyl sulfate
PAGE: polyacrylamide gel electrophoresis
RT-PCR: reverse transcription polymerase chain reaction
BIR: baculovirus IAP repeat
BIRCs: BIR domain-containing proteins
FBS: fetal bovine serum
TBS-T: Tris-buffered saline containing 0.05% Tween 20
HRP: horseradish peroxidase
GSK: glycogen synthase kinase
IHC: immunohistochemical
DAB: 3,3′-diaminobenzidine
LAS: Leica Application Suite
