# Supplementary material for: Cellular inhibitor of apoptosis protein 2 promotes the epithelial-mesenchymal transition in triple-negative breast cancer cells through activation of the AKT signaling pathway
Source: Oncotarget. 2017 Aug 12;8(45):78781–95. doi: 10.18632/oncotarget.20227 (PMC5667998; doi:10.18632/oncotarget.20227)
Supplement: Supplementary file 1 [file oncotarget-08-78781-s001.pdf]

Cellular inhibitor of apoptosis protein 2 promotes the epithelial-mesenchymal transition in triple-negative breast cancer cells through activation of the AKT signaling pathway

SUPPLEMENTARY MATERIALS

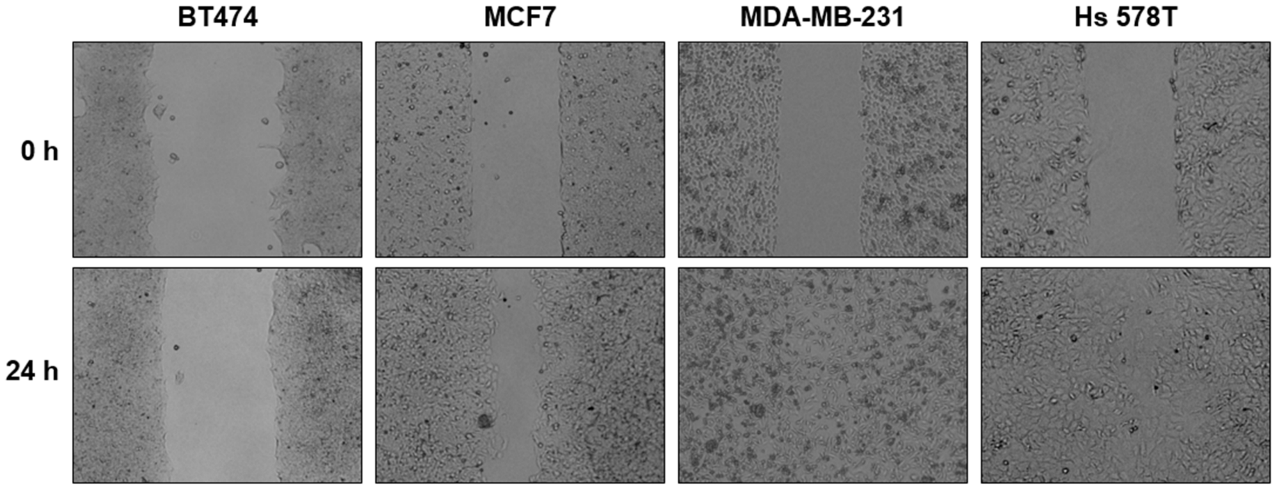

Supplementary Figure 1: Cell motility in various breast cancer cell lines (BT474, MCF7, MDA-MB-231, Hs 578T). Cell migration capacity was examined by wound healing assays. Images were taken at 0 and 24 h. The wound width was 500 μm (10× magnification).

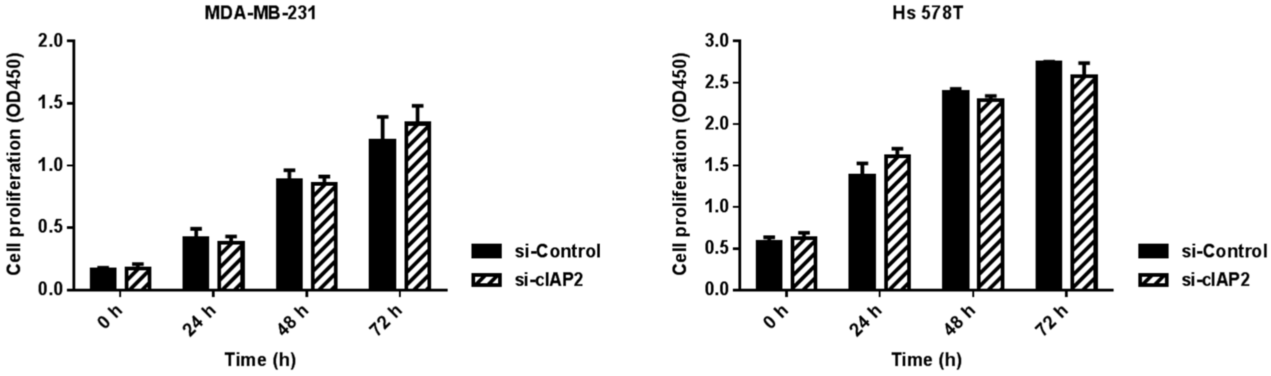

Supplementary Figure 2: Effect of cIAP2 silencing on cell proliferation in two TNBC cell lines (MDA-MB-231 and Hs 578T). Cell proliferation rates of the indicated cell lines were measured using a Cell Counting Kit-8 (CCK-8). Values are represented as means ± standard deviations.

Supplementary Table 1: Characteristics of the breast cancer cell lines used in this study

Cell line	ER	PR	HER2	Classification
MCF7	+	+	-	Luminal [18-20]
T-47D	+	+	-	Luminal [18-20]
BT474	+	+	+	Luminal [18-20]
SK-BR-3	-	-	+	HER2-positive[18-20]
MDA-MB-231	-	-	-	Triple-negative [18-20]
Hs 578T	-	-	-	Triple-negative [18-20]

Statuses of estrogen receptor (ER), progesterone receptor (PR), and human epidermal growth factor receptor 2 (HER2)/neu as well as breast cancer subtypes are indicated based on previously published data.

Supplementary Table 2: IHC staining scores

% Staining score	% Nuclei staining
0	0%
1	1–4%
2	5–9%
3	10–19%
4	> 20%
5	> 50%
**Intensity score**	**Staining intensity**
0	No staining
1	Weak staining
2	Moderate staining
3	Strong staining

Quick score = % Staining score + Intensity score
Giving a range from 0 to 8

Supplementary Table 3: Sequences of the primers used in this study

Gene product	Sense	Antisense
CDH1 **(E-cadherin)**	GTCAGTTCAGACTCCAGCCC	AAATTCACTCTGCCCAGGACG
EPCAM **(EpCAM)**	CTCCAGAACAATGATGGGCT	CCAGTAGGTTCTCACTCGCTC
CDH2 **(N-cadherin)**	GGTGGAGGAGAAGAAGACCAG	GGCATCAGGCTCCACAGT
Vim **(vimentin)**	GTTTCCAAGCCTGACCTCAC	GCTTCAACGGCAAAGTTCTC
ACTA2 **(α-SMA)**	GTGAAGAAGAGGACAGCACTG	CACATACATGGCTGGGACATTG
SNAI2 **(Slug)**	CAAGGACACATTAGAACTCAC	GTGCAGGAGAGACATTCTGGA
SNAI1 **(Snail)**	ACTTCAGTCTCTTCCTTGGAG	TGACATCTGAGTGGGTCTGG
BIRC1 **(NAIP)**	CGTCCCATTTGTTGCCAGT	GATCAGTTTGGCCACTCG
BIRC2 **(cIAP1)**	GTGGTGGGAAGCTCAGTAAC	CATCATCATTGCGACCCACA
BIRC3 **(cIAP2)**	AATGCTTTTGCTGTGATGGTG	GCTTGAACTTGACGGATGAAC
BIRC4 **(XIAP)**	GGGGTTCAGTTTCAAGGACAT	CCACAAGGAACAAAAACGATAG
BIRC5 **(survivin)**	GACGACCCCATAGAGGAACAT	GCCAGAGGCCTCAATCCAT
BIRC6 **(BRUCE)**	ATGTGCTTCCAACCCTTCCT	GGCTCAGTTTGATTCACGC
BIRC7 **(livin)**	CTGGGACCCGTGGGAAGAAC	TCCTGGGCACTTTCAGACTG
BIRC8 **(ILP2)**	GAGACGGTGGACAAGTCCTA	TTGCCACCTGTCTACCGCTT
GAPDH	GATGGCATGGACTGTGGTCA	GCAATGCCTCCTGCACCACC
